# Applying the international classification of diseases to perinatal mortality data, South Africa 

**DOI:** 10.2471/BLT.17.206631

**Published:** 2018-10-17

**Authors:** Tina Lavin, Emma R Allanson, Lee Nedkoff, David B Preen, Robert C Pattinson

**Affiliations:** a Centre for Health Services Research, School of Population and Global Health, The University of Western Australia, 35 Stirling Highway, Crawley WA 6009, Perth, Australia.; b School of Women’s and Infants’ Health, The University of Western Australia, Perth, Australia.; c Cardiovascular Research Group, School of Population and Global Health, The University of Western Australia, Perth, Australia.; d Maternal and Infant Health Care Strategies Unit, University of Pretoria, Pretoria, South Africa.

## Abstract

**Objective:**

To examine the feasibility of applying the International Classification of Diseases-perinatal mortality (ICD-PM) coding to an existing data set in the classification of perinatal deaths.

**Methods:**

One author, a researcher with a non-clinical public health background, applied the ICD-PM coding system to South Africa’s national perinatal mortality audit system, the Perinatal Problem Identification Program. The database for this study included all perinatal deaths (*n* = 26 810), defined as either stillbirths (of birth weight > 1000 g and after 28 weeks of gestation) or early neonatal deaths (age 0–7 days), that occurred between 1 October 2013 and 31 December 2016. A clinical obstetrician verified the coding.

**Findings:**

The South African classification system does not include the timing of death; however, under the ICD-PM system, deaths could be classified as antepartum (*n* = 15 619; 58.2%), intrapartum (*n* = 3725; 14.0%) or neonatal (*n* = 7466; 27.8%). Further, the South African classification system linked a maternal condition to only 40.3% (10 802/26 810) of all perinatal deaths; this proportion increased to 68.9% (18 467/26 810) under the ICD-PM system.

**Conclusion:**

The main benefit of using the clinically relevant and user-friendly ICD-PM system was an enhanced understanding of the data, in terms of both timing of death and maternal conditions. We have also demonstrated that it is feasible to convert an existing perinatal mortality classification system to one which is globally comparable and can inform policy-makers internationally.

## Résumé

**Objectif:**

Examiner s'il est faisable d'appliquer les codes de la Classification internationale des maladies pour la période périnatale (ICD-PM) à un ensemble de données existantes de classification des décès périnatals.

**Méthodes:**

L'une des auteurs de cette publication, une chercheuse ayant une expérience non clinique en santé publique, a appliqué le système de codage ICD-PM au système d'audit national de la mortalité périnatale d'Afrique du Sud, le *Perinatal Problem Identification Program*. La base de données utilisée pour cette étude incluait l'intégralité des décès périnatals (*n* = 26 810) survenus entre le 1^er^ octobre 2013 et le 31 décembre 2016 et définis soit comme des mortinaissances (poids de naissance > 1 000 g et 28 semaines de gestation révolues), soit comme des décès néonatals précoces (entre l'âge de 0 à 7 jours). Une clinicienne-obstétricienne a vérifié ce codage.

**Résultats:**

Le système de classification d'Afrique du Sud ne consigne pas le moment d’intervention du décès, néanmoins, avec le système ICD-PM, les décès ont pu être classés en tant que décès fœtaux (*n* = 15 619; 58,2%), décès per-partum (*n* = 3 725; 14,0%) ou décès néonatals (*n* = 7 466; 27,8%). Par ailleurs, dans le système de classification d'Afrique du Sud, seuls 40,3% (10 802/26 810) des décès périnatals avaient été reliés à une affection maternelle; une proportion qui est passée à 68,9% (18 467/26 810) avec le système ICD-PM.

**Conclusion:**

Le principal avantage obtenu avec l'utilisation du système ICD-PM, par ailleurs facile d’emploi et cliniquement pertinent, a été de permettre une meilleure compréhension des données, à la fois concernant le moment du décès et concernant les affections maternelles. Nous avons également démontré qu'il était faisable de convertir un système de classification de la mortalité périnatale existant vers un système qui permet d'obtenir des données comparables internationalement et susceptibles d'éclairer les décisions politiques à l'échelle mondiale.****

## Resumen

**Objetivo:**

Examinar la viabilidad de aplicar la codificación de la Clasificación Internacional de Enfermedades-Mortalidad Perinatal (CIE-MP) a un conjunto de datos existente en la clasificación de muertes perinatales.

**Métodos:**

Un autor, un investigador con formación no clínica en salud pública, aplicó el sistema de codificación CIE-MP al sistema nacional de auditoría de mortalidad perinatal de Sudáfrica, el Programa de Identificación de Problemas Perinatales. La base de datos para este estudio incluyó todas las muertes perinatales (n = 26 810), definidas como mortinatos (con un peso al nacer > 1000 g y después de 28 semanas de gestación) o muertes neonatales tempranas (de 0 a 7 días de edad), que tuvieron lugar entre el 1 de octubre de 2013 y el 31 de diciembre de 2016. Un obstetra clínico verificó el código.

**Resultados:**

El sistema de clasificación sudafricano no incluye el momento de la muerte; sin embargo, con el sistema CIE-MP, las muertes se pueden clasificar como anteparto (n = 15 619; 58,2 %), intraparto (n = 3725; 14,0 %) o neonatales (n = 7466; 27,8 %). Además, el sistema de clasificación sudafricano vinculó una afección materna con solo el 40,3 % (10 802/26 810) de todas las muertes perinatales; esta proporción aumentó al 68,9 % (18 467/26 810) en el marco del sistema CIE-MP.

**Conclusión:**

El principal beneficio de utilizar el clínicamente relevante y fácil de usar sistema CIE-MP fue la mejor comprensión de los datos, tanto en lo relativo al momento de la muerte como a las condiciones maternas. También se ha demostrado que es factible convertir un sistema existente de clasificación de mortalidad perinatal en uno que sea globalmente comparable y que pueda informar a los responsables de la formulación de políticas a nivel internacional.

## ملخص

الغرض التحقق من جدوى تطبيق نظام ترميز التصنيف الدولي للوفيات المرتبطة بالأمراض المحيطة بالولادة (ICD-PM) على مجموعة بيانات موجودة في تصنيف حالات الوفاة في الفترة المحيطة بالولادة.

الطريقة قام أحد المؤلفين، وهو باحث لديه خلفية صحية عامة غير إكلينيكية، بتطبيق نظام الترميز في تصنيف ICD-PM، على نظام مراجعة الوفيات الوطني في الفترة المحيطة بالولادة في جنوب أفريقيا، وهو برنامج تحديد المشكلة خلال الفترة المحيطة بالولادة.تضمنت قاعدة بيانات هذه الدراسة جميع وفيات الفترة المحيطة بالولادة (العدد = 26810)، التي تم تحديدها إما وفيات للرُضّع (وزن الولادة > 1000 جرام وبعد 28 أسبوعًا من الحمل)، أو وفيات مبكرة للمواليد (عند عمر 0 إلى 7 أيام)، والتي حدثت بين 1 أكتوبر/تشرين أول 2013 و31 ديسمبر/كانون أول 2016. وقام طبيب سريري للتوليد بالتحقق من الترميز.

النتائج لا يتضمن نظام التصنيف في جنوب أفريقيا توقيت الوفاة؛ ومع ذلك، فإنه بموجب نظام ICD-PM، يمكن تصنيف الوفيات على أنها حالات قبل الولادة (العدد = 15619؛ بنسبة 58.2٪)، أو حالات أثناء الولادة (العدد = 3725؛ 14.0٪) أو حالات بعد الولادة (العدد = 7466؛ 27.8٪).وعلاوة على ذلك، ربط نظام التصنيف في جنوب أفريقيا بين حالة الأم وبين 40.3٪ فقط (10802/26810) من جميع وفيات الفترة المحيطة بالولادة؛ وارتفعت هذه النسبة إلى 68.9٪ (18467/26810) بموجب نظام ICD-PM.

الاستنتاج يتميز نظام ICD-PM بسهولة الاستخدام والملاءمة للجوانب السريرية، وتتمثل الفائدة الرئيسية لاستخدامه في تعزيز فهم البيانات، فيما يتعلق بكل من توقيت الوفاة وحالة الأم.كما أوضحنا أنه من الممكن تحويل نظام تصنيف الوفيات في الفترة المحيطة بالولادة إلى نظام قابل للمقارنة بشكل عالمي، ويمكن أن يفيد واضعي السياسات على المستوى الدولي.

## 摘要

**目的:**

旨在检验对围产儿死亡率的现有数据集应用国际疾病分类法-围产儿死亡率 (ICD-PM) 编码的可行性。

**方法:**

笔者之一,同时也是一位具有非临床公共卫生背景的研究人员,将 ICD-PM 编码系统应用于南非全国性围产儿死亡率审计系统,即围产儿问题识别计划。该项研究的数据库包括 2013 年 10 月 1 日至 2016 年 12 月 31 日的所有围产儿死亡案例(案例数量为 26810 例),定义为死产儿(出生时体重大于 1000 克,妊娠期 28 周后)或早期新生儿死亡(出生 0-7 天内死亡)。临床产科医生验证了编码。

**结果:**

南非分类系统不涉及死亡时间;然而,在 ICD-PM 系统下,死亡案例可根据死亡时间划分为产前死亡(案例数量为 15619 例;占 58.2%),分娩时死亡(案例数量为 3725 例;占 14.0%)或新生儿死亡(案例数量为 7466 例;占 27.8%)。此外,南非分类系统将孕产妇状况仅与 40.3% 的围产儿死亡(26810 例中占 10802 例)联系起来;在 ICD-PM 系统下,这一比例增加至 68.9%(26810 例中占 18467 例)。

**结论:**

使用临床相关且用户友好的 ICD-PM 系统的主要益处在于加强了对死亡时间与孕产妇状况两方面数据的理解。同时证明,转换现有围产儿死亡率分类系统为全球可比系统是可行的,并可为全球范围内的决策制定者提供信息。

## Резюме

**Цель:**

Изучить возможность применения кодирования МКБ-ПС (Международная классификация болезней — перинатальная смертность) к существующему набору данных в классификации перинатальной смертности.

**Методы:**

Один автор, проводивший доклинические исследования в области общественного здравоохранения, применил систему кодирования МКБ-ПС к Национальной системе аудита перинатальной смертности в Южной Африке, Программе выявления заболеваний, возникающих в перинатальном периоде. База данных для этого исследования включала все случаи смерти в перинатальном периоде (*n* = 26 810), определяемые как мертворождение (вес при рождении > 1000 г и роды после 28 недель беременности) или смерть в раннем неонатальном периоде (возраст 0–7 дней), которые были зарегистрированы с 1 октября 2013 года по 31 декабря 2016 года. Проверка кодирования осуществлялась клиническим акушером.

**Результаты:**

Южноафриканская система классификации не учитывает время наступления смерти, однако в рамках системы МКБ-ПС смерть может быть классифицирована как антенатальная (*n* = 15 619, 58,2%), интранатальная (*n* = 3725, 14,0%) или неонатальная (*n* = 7466, 27,8%). Кроме того, южноафриканская система классификации связывала состояние здоровья матери лишь с 40,3% (10 802/26 810) от всех случаев перинатальной смерти. При использовании системы МКБ-ПС эта доля увеличилась до 68,9% (18 467/26 810).

**Вывод:**

Основным преимуществом применения клинически значимой и удобной в использовании системы МКБ-ПС является более глубокое понимание таких аспектов, как время наступления смерти и состояние здоровья матери. Исследование также продемонстрировало, что на основе существующей системы классификации перинатальной смертности можно создать усовершенствованную систему, обеспечивающую сопоставимость на глобальном уровне и информирующую высокопоставленных должностных лиц по всему миру.

## Introduction

High on the global health agenda is the need to accelerate progress towards ending preventable perinatal deaths, defined by the World Health Organization (WHO) as either a stillbirth of weight > 1000 g or after at least 28 weeks gestation, or an early neonatal death in the first 7 days after birth.^1^ In developing appropriate intervention strategies to reach this target, the causes of perinatal deaths must be classified in a globally comparable way.^2^^,^^3^ A recent systematic review identified no less than 81 different systems used to classify perinatal deaths globally, with only 17 systems using the *International Statistical Classification of Diseases and Related Health Problems* (ICD) codes.^4^ Other studies have recognized that multiple, disparate systems impede the ability to understand and achieve accurate estimates of cause of death, hindering effective prevention strategies.^5^^,^^6^ Of particular importance is the need to focus on the mother–infant dyad, as maternal condition is closely related to perinatal death.^1^

The Every Newborn Action Plan recommends that maternal complications be recorded as part of perinatal death registration; however, challenges existed in applying the 10th edition of the ICD (ICD-10) classification system as maternal condition was not linked to perinatal condition.^7^ To address these issues, the WHO application of ICD-10 to perinatal deaths (ICD-perinatal mortality or ICD-PM) was published in 2016,^8^^,^^9^ the first perinatal death classification system developed for application globally.^10^ ICD-PM is modelled on the WHO application of the ICD-10 system to deaths during pregnancy, childbirth and the puerperium (ICD-maternal mortality or ICD-MM),^11^ and follows all coding rules of ICD-10.^12^ Importantly, the ICD-PM system identifies the timing of perinatal death (i.e. antepartum, intrapartum or neonatal), links causes of death to existing ICD-10 codes and connects maternal condition with perinatal death.^8^ One of the aims of ICD-PM is to group ICD-10 codes into clinically relevant and easy-to-use categories.^10^

We demonstrate the benefits achieved, in terms of an improved understanding of the data, from the application of ICD-PM codes to perinatal deaths that were previously classified using the South African perinatal mortality audit system, called Perinatal Problem Identification Program.

## Methods

### Data source

South Africa’s perinatal mortality audit system^13^ records and classifies perinatal deaths at all 588 clinics across the country. Each clinical team performs a mortality review shortly after death and reports the cause of perinatal death (and associated maternal condition) to the classification system. For the purposes of the system, perinatal deaths are defined as either fresh or macerated stillbirth or early neonatal death (age 0–7 days). The primary obstetric cause of death is classified in terms of both lead categories and subcategories according to Box 1. Maternal condition is also recorded, and classified as either healthy (where the examining clinician did not identify any clinical problems) or as one of the medical/obstetric conditions listed in Box 2. Classifications of perinatal death are linked to maternal condition lead categories, but not to subcategories. Data are joined into a national database at the Medical Research Council Unit for Maternal and Infant Health Care Strategies, Pretoria. Regular auditing of individual clinics is conducted to ensure the completeness and accuracy of the database.

Box 1 **South African Perinatal Problem Identification Program classification of primary cause of perinatal death**

**Intrauterine death**

Subcategories: unexplained intrauterine death (macerated); unexplained intrauterine death (fresh); and unexplained intrauterine death (due to lack of notes)

**Intrapartum asphyxia**

Subcategories: Labour-related intrapartum asphyxia; meconium aspiration; cord around the neck; cord prolapsed; ruptured uterus; traumatic breech delivery; shoulder dystocia; precipitous labour; and traumatic assisted delivery

**Hypertensive disorders**

Subcategories: proteinuric hypertension; eclampsia; pregnancy-induced hypertension without proteinuria; and chronic hypertension

**Antepartum haemorrhage**

Subcategories: abruptio placentae; abruptio placentae with hypertension; antepartum haemorrhage of unknown origin; and placenta praevia

**Spontaneous preterm labour**

Subcategories: idiopathic preterm labour; premature rupture of membranes; iatrogenic preterm delivery for no real reason; premature rupture of membranes with chorioamnionitis; preterm labour with chorioamnionitis with intact membranes; and cervical incompetence

**Fetal abnormality**

Subcategories: fetal chromosomal abnormality; abnormality of multiple systems; neural tube defects; hydrocephalus; non-specific fetal abnormality; cardiovascular system abnormality; non-immune hydrops fetalis; and renal system abnormality

**Infections**

Subcategories: other infections; amniotic fluid infection; syphilis; β-haemolytic streptococcal infection; and malaria

**Intrauterine growth restriction**

Subcategories: idiopathic intrauterine growth restriction; postmaturity; and with histological features of ischaemic placental disease

**No obstetric cause**

**Maternal disease**

Subcategories: maternal diabetes mellitus; other maternal disease; maternal disease due to herbal medicine use; and maternal heart disease

**Miscellaneous**

Subcategories: other cause of death not described in classification; twin-to-twin transfusion; rhesus isoimmunization; and extrauterine pregnancy

**Trauma**

Subcategories: motor vehicle accident; accidental abdominal trauma; assault; and domestic violence

Box 2 **South African Perinatal Problem Identification Program classification of maternal conditions**

**No obstetric condition**

Subcategory: healthy mother

**Coincidental conditions**

Subcategories: herbal medicine; other coincidental conditions; other accidents; motor vehicle accident; assault; and rape

**Medical and surgical disorders**

Subcategories: endocrine disease; other medical and surgical disorders; autoimmune disease; haematological disease; genitourinary disease; cardiac disease; respiratory disease; central nervous system disease; psychiatric disease; gastrointestinal disease; neoplastic disease; and skeletal disease

**Non-pregnancy-related infections**

Subcategories: urinary tract infection; other non-pregnancy-related infections; tuberculosis; complications of antiretroviral therapy; wasting syndrome; other pneumonia; pneumocystis pneumonia; malaria; gastroenteritis; other meningitis; cryptococcal meningitis; hepatitis; Kaposi sarcoma; appendicitis; endocarditis; and toxoplasmosis

**Pregnancy-related sepsis**

Subcategories: chorioamnionitis with ruptured membranes; and chorioamnionitis with intact membranes

**Obstetric haemorrhage**

Subcategories: abruption with hypertension; abruption without hypertension; other acute postoperative hypertension (not specified); placenta praevia; ruptured uterus with previous caesarean section; and ruptured uterus without previous caesarean section

**Hypertension**

Subcategories: Proteinuric hypertension; pregnancy-induced hypertension without proteinuria; eclampsia; chronic hypertension; haemolysis, elevated liver enzymes and low platelet count (HELLP syndrome); liver rupture; and acute fatty liver

**Acute collapse (cause unknown)**

**Anaesthetic complications**

Subcategories: complications of general anaesthetic; complications of spinal anaesthetic; complications of epidural anaesthetic

**Extrauterine pregnancy**

**Embolism**

Subcategories: amniotic fluid embolism; and pulmonary embolism

Note: Only lead categories and not subcategories can be linked to perinatal deaths.

We used all 26 810 perinatal deaths, which occurred during the period between 1 October 2013 and 31 December 2016, recorded in the classification system’s database (Table 1). The start date coincided with the launch of the third version of the system, which had been improved to include gestational age at death. 

**Table 1:** Primary cause of perinatal death as classified by South Africa’s Perinatal Problem Identification Program, 1 October 2013 and 31 December 2016

Condition	No. of perinatal deaths (%)													
	Primary cause												Total	
	Unexplained intrauterine death	Intrapartum asphyxia	Hypertensive disorders	Antepartum haemorrhage	Spontaneous preterm labour	Fetal abnormality	Infections	Intrauterine growth restriction	No obstetric cause	Maternal disease	Miscellaneous	Trauma			
**No maternal condition**														
Fresh stillbirths	632 (19.3)	1478 (45.1)	56 (1.7)	370 (11.3)	263 (8.0)	252 (7.7)	55 (1.7)	76 (2.3)	32 (1.0)	13 (0.4)	40 (1.2)	11 (0.3)		3278 (100)	
Macerated stillbirths	4729 (67.5)	421 (6.0)	252 (3.6)	206 (2.9)	384 (5.5)	263 (3.8)	162 (2.3)	335 (4.8)	68 (1.0)	69 (1.0)	98 (1.4)	18 (0.3)		7005 (100)	
Early neonatal deaths	0 (0.0)	2430 (42.4)	53 (0.9)	105 (1.8)	1772 (31.0)	661 (11.5)	139 (2.4)	91 (1.6)	345 (6.0)	11 (0.2)	110 (1.9)	8 (0.1)		5725 (100)	
**With maternal cause of death**														
Coincidental conditions														
Fresh stillbirths	25 (16.9)	67 (45.3)	6 (4.1)	13 (8.8)	5 (3.4)	3 (2.0)	5 (3.4)	3 (2.0)	1 (0.7)	3 (2.0)	1 (0.7)	16 (10.8)		148 (100)	
Macerated stillbirths	93 (52.2)	17 (9.6)	8 (4.5)	7 (3.9)	9 (5.1)	4 (2.2)	12 (6.7)	3 (1.7)	0 (0.0)	9 (5.1)	3 (1.7)	13 (7.3)		178 (100)	
Early neonatal deaths	0 (0.0)	76 (42.7)	6 (3.4)	8 (4.5)	40 (22.5)	9 (5.1)	11 (6.2)	1 (0.6)	15 (8.4)	3 (1.7)	7 (3.9)	2 (1.1)		178 (100)	
Medical and surgical disorders															
Fresh stillbirths	49 (15.3)	78 (24.4)	26 (8.1)	76 (23.8)	12 (3.8)	13 (4.1)	14 (4.4)	2 (0.6)	1 (0.3)	37 (11.6)	3 (0.9)	9 (2.8)		320 (100)	
Macerated stillbirths	307 (34.5)	30 (3.4)	112 (12.6)	58 (6.5)	27 (3.0)	22 (2.5)	50 (5.6)	25 (2.8)	1 (0.1)	243 (27.3)	12 (1.3)	2 (0.2)		889 (100)	
Early neonatal deaths	0 (0.0)	87 (25.1)	21 (6.1)	15 (4.3)	97 (28.0)	32 (9.2)	27 (7.8)	9 (2.6)	18 (5.2)	26 (7.5)	13 (3.8)	1 (0.3)		346 (100)	
Non-pregnancy-related infections															
Fresh stillbirths	29 (21.2)	51 (37.2)	3 (2.2)	11 (8.0)	10 (7.3)	4 (2.9)	22 (16.1)	3 (2.2)	0 (0.0)	1 (0.7)	1 (0.7)	2 (1.5)		137 (100)	
Macerated stillbirths	191 (50.1)	11 (2.9)	26 (6.8)	13 (3.4)	26 (6.8)	11 (2.9)	65 (17.1)	23 (6.0)	4 (1.0)	10 (2.6)	1 (0.3)	0 (0.0)		381 (100)	
Early neonatal deaths	0 (0.0)	66 (25.2)	9 (3.4)	11 (4.2)	79 (30.2)	18 (6.9)	38 (14.5)	13 (5.0)	18 (6.9)	6 (2.3)	4 (1.5)	0 (0.0)		262 (100)	
Pregnancy-related sepsis															
Fresh stillbirths	2 (3.1)	12 (18.8)	0 (0.0)	4 (6.3)	13 (20.3)	4 (6.3)	28 (43.8)	0 (0.0)	0 (0.0)	0 (0.0)	1 (1.6)	0 (0.0)		64 (100)	
Macerated stillbirths	17 (21.0)	1 (1.2)	3 (3.7)	2 (2.5)	19 (23.5)	1 (1.2)	34 (42.0)	1 (1.2)	1 (1.2)	1 (1.2)	1 (1.2)	0 (0.0)		81 (100)	
Early neonatal deaths	0 (0.0)	18 (19.8)	1 (1.1)	0 (0.0)	45 (49.5)	5 (5.5)	18 (19.8)	2 (2.2)	1 (1.1)	0 (0.0)	1 (1.1)	0 (0.0)		91 (100)	
Obstetric haemorrhage															
Fresh stillbirths	38 (2.5)	154 (10.0)	60 (3.9)	1239 (80.6)	9 (0.6)	4 (0.3)	4 (0.3)	2 (0.1)	1 (0.1)	2 (0.1)	2 (0.1)	22 (1.4)		1537 (100)	
Macerated stillbirths	48 (5.9)	21 (2.6)	61 (7.4)	664 (81.0)	6 (0.7)	1 (0.1)	1 (0.1)	3 (0.4)	3 (0.4)	3 (0.4)	0 (0.0)	9 (1.1)		820 (100)	
Early neonatal deaths	0 (0.0)	27 (9.4)	3 (1.0)	225 (78.1)	19 (6.6)	7 (2.4)	1 (0.3)	0 (0.0)	1 (0.3)	0 (0.0)	0 (0.0)	5 (1.7)		288 (100)	
Hypertension															
Fresh stillbirths	59 (5.1)	182 (15.7)	606 (52.2)	246 (21.2)	11 (0.9)	20 (1.7)	3 (0.3)	17 (1.5)	2 (0.2)	9 (0.8)	3 (0.3)	4 (0.3)		1162 (100)	
Macerated stillbirths	286 (10.6)	67 (2.5)	1965 (72.9)	187 (6.9)	22 (0.8)	21 (0.8)	12 (0.4)	55 (2.0)	2 (0.1)	58 (2.2)	15 (0.6)	4 (0.1)		2694 (100)	
Early neonatal deaths	0 (0.0)	230 (23.8)	501 (51.9)	30 (3.1)	65 (6.7)	67 (6.9)	14 (1.4)	20 (2.1)	20 (2.1)	10 (1.0)	9 (0.9)	0 (0.0)		966 (100)	
Other maternal condition^a^															
Fresh stillbirths	14 (25.0)	8 (14.3)	5 (8.9)	9 (16.1)	4 (7.1)	1 (1.8)	2 (3.6)	2 (3.6)	2 (3.6)	2 (3.6)	6 (10.7)	1 (1.8)		56 (100)	
Macerated stillbirths	87 (49.2)	8 (4.5)	25 (14.1)	5 (2.8)	14 (7.9)	1 (0.6)	4 (2.3)	3 (1.7)	0 (0.0)	7 (4.0)	21 (11.9)	2 (1.1)		177 (100)	
Early neonatal deaths	1 (3.7)	8 (29.6)	4 (14.8)	1 (3.7)	4 (14.8)	2 (7.4)	1 (3.7)	0 (0.0)	2 (7.4)	2 (7.4)	2 (7.4)	0 (0.0)		27 (100)	

^a^ Extrauterine pregnancy, anaesthetic complications, embolism and acute collapse combined.

### Conversion to ICD-PM coding

The first author, a non-clinical researcher with a background in public health, studied *The WHO application of ICD-10 to deaths during the perinatal period: ICD-PM*^9^ to learn to apply ICD-PM codes to the classification system’s database. The coding conversion took place between November 2017 and January 2018. The second author, a consulting obstetrician, provided guidance and verification on a voluntary basis. The ICD-PM system^2^^,^^9^^,^^14^ classifies mortality according to: (i) time of death, whether antepartum (A1–A6), intrapartum (I1–I7) or neonatal (N1–N11); (ii) the primary cause of perinatal death (e.g. loss of fetal blood: P50); and (iii) the main maternal condition (M1–M4 to describe various complications and conditions, and M5 for healthy mother) at the time of perinatal death.

### Ethics

Data were collected with the permission of the South African Department of Health. This analysis was approved by the technical task team who run the database and produce the reports from the South African Medical Research Council/University of Pretoria Maternal and Infant Health Care Strategies unit. This was a secondary analysis and all identifiers of the cases were removed. Ethics approval was given by the University of Western Australia Human Ethics Committee (RA/4/1/7955, 20 November 2015).

## Results

Table 2 shows the reclassification of perinatal deaths using the ICD-PM and the primary causes of death are linked to maternal condition. Most deaths were antepartum in timing (15 619/26 810; 58.3%), followed by neonatal (7466/26 810; 27.8%) and intrapartum (3725/26 810; 13.9%). Of the total number of perinatal deaths, 8.8% (2368) were associated with a maternal death.

**Table 2:** Application of ICD-PM codes to perinatal deaths recorded in South African Perinatal Problem Identification Program, 1 October 2013–31 December 2016

Perinatal condition	Maternal condition^a^					Total (%)
	M1	M2	M3	M4	M5	
**No. of antepartum deaths**						
A1: Congenital malformations, deformations and chromosomal abnormalities	5	0	0	75	334	414 (2.7)
A2: Infection	83	1	0	310	52	446 (2.9)
A3: Antepartum hypoxia	0	0	0	0	0	0 (0.0)
A4: Other specified antepartum disorder	2 342	10	0	595	0	2 947 (18.9)
A5: Disorders related to fetal growth	122	518	0	283	347	1 270 (8.1)
A6: Fetal death of unspecified cause	246	45	0	4573	5678	10 542 (67.5)
Total (% of antepartum deaths)	2 798 (17.9)	574 (3.7)	0 (0.0)	5836 (37.4)	6411 (41.0%)	15 619 (100.0)
**No. of intrapartum deaths**						
I1: Congenital malformations, deformations and chromosomal abnormalities	0	0	0	29	161	190 (5.1)
I2: Birth trauma	0	0	0	0	0	0 (0.0)
I3: Acute intrapartum event	919	8	932	518	199	2 576 (69.2)
I4: Infection	11	0	0	32	0	43 (1.2)
I5: Other specified intrapartum disorder	350	52	0	77	0	479 (12.9)
I6: Disorders related to fetal growth	15	1	0	20	28	64 (1.7)
I7: Intrapartum death of unspecified cause	4	9	0	337	23	373 (10.0)
Total (% of intrapartum deaths)	1 299 (34.9)	70 (1.9)	932 (25.0)	1 013 (27.2)	411 (11.0)	3 725 (100.0)
**No. of neonatal deaths**						
N1: Congenital malformations, deformations and chromosomal abnormalities	7	1	0	133	583	724 (9.7)
N2: Disorders related to fetal growth	14	0	0	45	77	136 (1.8)
N3: Birth trauma	0	0	0	0	0	0 (0.0)
N4: Complications of intrapartum events	200	1	1 660	323	0	2 184 (29.3)
N5: Convulsions and disorders of cerebral status	0	0	0	0	0	0 (0.0)
N6: Infection	50	2	0	164	54	270 (3.6)
N7: Respiratory and cardiovascular disorders	4	0	0	756	674	1 434 (19.2)
N8: Other neonatal conditions	334	2	0	79	0	415 (5.6)
N9: Low birth weight and prematurity	51	226	1 458	331	62	2 128 (28.5)
N10: Miscellaneous	5	3	0	96	71	175 (2.3)
N11: Neonatal death of unspecified cause	0	0	0	0	0	0 (0.0)
Total (% of neonatal deaths)	665 (8.9)	235 (3.1)	3 118 (41.8)	1 927 (25.8)	1 521 (20.4)	7 466 (100.0)

ICD-PM: International Classification of Diseases-perinatal mortality.

^a^ M1: complications of placenta, cord and membranes; M2: maternal complications of pregnancy; M3: other complications of labour and delivery; M4: maternal medical and surgical conditions; M5: no maternal condition.

### Antepartum deaths

Antepartum deaths were largely due to fetal deaths of an unspecified cause (10 542 deaths; 67.5%; ICD-PM code A6), other specified antepartum disorder (2947 deaths; 18.9%; A4) or disorders related to fetal growth (1270 deaths; 8.1%; A5; Table 2). Of the 15 619 antepartum deaths recorded, 41.0% (6411) of the mothers had no maternal condition. For most of antepartum deaths classified as fetal death of unspecified cause, the mothers were free from any maternal complication (53.9%; 5678/10 542; A6 M5): 5537 (97.5%) deaths were due to an unexplained intrauterine death (A6 P95), 56 deaths (1.0%) were described as miscellaneous or other causes not described by the South African classification (A6 P95), and 85 (1.5%) deaths had no obstetric cause (M5).

Antepartum deaths classified as other specified antepartum disorder (2947 deaths; 18.9%; A4) were further classified under fetal blood loss (2342 deaths; P50), with the main causes being abruptio placentae (1124 deaths; 38.1%; A4 P50 M1 P02.1), abruptio placentae with hypertension (928 deaths; 31.5%; A4 P50 M1 P02.1), antepartum haemorrhage of unknown origin (106 deaths; 3.6%; A4 P50 M1 P02.1), placenta praevia (69 deaths; 2.3%; A4 P50 M1 P02.0) and twin-to-twin transfusion (115 deaths; 3.9%; A4 P50 M1 P02.3). Where fetal blood loss was the primary cause of perinatal death, 595 deaths were related to maternal medical and surgical conditions (M4), including: hypertension (406 deaths), medical and surgical complications (114 deaths), non-pregnancy-related infections (21 deaths), coincidental (14 deaths), sepsis (4 deaths), anaesthetic (4 deaths) and acute collapse (3 deaths), all coded as A4 P50 M4; and rhesus isoimmunization (29 deaths), coded as A4 P55.0 M4 P00.9. There were 10 antepartum haemorrhages with ectopic pregnancies, coded as A4 P50 M2 P01.4. Causes of death relating to haemorrhage described by code P52 are not categorized in the South African database, so we could not utilize this code during our conversion.

### Intrapartum deaths

The main causes of the 3725 intrapartum deaths (Table 2) were acute intrapartum event (2576 deaths; 69.2%; I3), other specified intrapartum disorder (479 deaths; 12.9%; I5) or intrapartum death of unspecified cause (373 deaths; 10.0%; I7). The other 297 deaths were classified as congenital malformations, deformations and chromosomal abnormalities (190 deaths; 5.1%; I1), infection (43 deaths; 1.7%; I4) and disorders related to fetal growth (64 deaths; 1.7%; I6). All deaths due to acute intrapartum events were classified as I3 P20.10 intrauterine hypoxia, first noted during labour and delivery. Of the intrapartum deaths of unspecified cause, most were due to hypertensive disorders (269 deaths; 72.1%) or maternal disease (46 deaths; 12.3%). Of all the intrapartum deaths; 11.0% (411 deaths) were not associated with any maternal complication.

### Neonatal deaths

The main causes of the 7466 neonatal deaths were complications of intrapartum events (2184 deaths; 29.3%; N4) or low birth weight and prematurity (2128 deaths; 28.5%; N9). All neonatal deaths classified as complications of intrapartum events were due to severe birth asphyxia (N4 P21; Table 2). Of all neonatal deaths, 1521 (20.4%) were not associated with any maternal complication. A large proportion (3118 deaths; 41.8%) of neonatal deaths were attributed to other complications of labour and delivery (M3). Of those deaths coded as M3, 1550 (49.7%) were due to labour-related intrapartum asphyxia (P03.9), 1456 (46.7%) were due to idiopathic preterm labour (P03.8 O60.0), 31 (1.0%) occurred as a result of traumatic breech delivery (P03.0), 25 (0.8%) were due to a precipitous labour (P03.5), 25 (0.8%) resulted from shoulder dystocia (P03.8 O66.0), 19 (0.6%) were due to traumatic assisted delivery (P03.2, P03.3) and 12 (0.4%) were due to a ruptured uterus (P03.8 O71.1).

Table 3 provides an example of how ICD-PM codes were applied to neonatal deaths classified by the South African classification system as being due to preterm labour. According to the classification system, most (83.5%, 1772/2121) of the deaths due to preterm labour were associated with a healthy maternal condition. Under the ICD-PM classification, however, 96.5% of these (1710/1772) were associated with a non-healthy maternal condition. For example, cases of perinatal death due to idiopathic preterm labour, premature rupture of membranes, premature rupture of membranes with chorioamnionitis, cervical incompetence and premature rupture of membranes with chorioamnionitis and intact membranes were assigned the codes M3 P03.8, M2 P01.1, M2 P01.1, M2 P01.0 and M1 P02.7, respectively. Only 3.5% (62/1772; iatrogenic preterm delivery for no real reason) of neonatal deaths due to preterm labour associated with a healthy mother, according to the South African classification, are coded as M5 under the ICD-PM system.

**Table 3:** Application of ICD-PM codes to classify spontaneous preterm labour neonatal deaths^a^ recorded in South African Perinatal Problem Identification Program, 1 October 2013–31 December 2016

Maternal condition	Perinatal condition as classified by Perinatal Problem Identification Program											
	Idiopathic preterm labour		Premature rupture of membranes		Iatrogenic preterm delivery for no real reason		Premature rupture of membranes with chorioamnionitis		Cervical incompetence		Preterm labour with chorioamnionitis with intact membranes	
	ICD-PM code	No. (%)	ICD-PM code	No. (%)	ICD-PM code	No. (%)	ICD-PM code	No. (%)	ICD-PM code	No. (%)	ICD-PM code	No. (%)
Healthy	N9 M3 P03.8	1458 (68.7)	N9 M2 P01.1	205 (9.7)	N9 M5	62 (2.9)	N9 M2 P01.1	21 (1.0)	N9 M2 P01.0	15 (0.7)	N9 M1 P02.7	11 (0.5)
Coincidental conditions	N9 M4 P00.5	33 (1.6)	N9 M4 P00.5	6 (0.3)	N9 M4 P00.5	1 (0.1)	N9 M4 P00.5	0 (0.0)	N9 M4 P00.5	0 (0.0)	N9 M4 P00.5	0 (0.0)
Medical and surgical disorders^b^	N9 M4 P00.0–P00.9	67 (3.2)	N9 M4 P00.0–P00.9	21 (1.0)	N9 M4 P00.0–P00.9	2 (0.1)	N9 M4 P00.0–P00.9	3 (0.1)	N9 M4 P00.0–P00.9	3 (0.1)	N9 M4 P00.0–P00.9	1 (0.1)
Non-pregnancy-related infection	N9 M4 P00.2	61 (2.9)	N9 M4 P00.2	6 (0.3)	N9 M4 P00.2	3 (0.1)	N9 M4 P00.2	3 (0.1)	N9 M4 P00.2	2 (0.1)	N9 M4 P00.2	4 (0.2)
Extrauterine pregnancy	N9 M2 P01.4	1 (0.1)	N9 M2 P01.4	1 (0.1)	N9 M2 P01.4	0 (0.0)	N9 M2 P01.4	0 (0.0)	N9 M2 P01.4	0 (0.0)	N9 M2 P01.4	0 (0.0)
Pregnancy-related sepsis	N9 M1 P02.7	9 (0.4)	N9 M1 P02.7	9 (0.4)	N9 M1 P02.7	2 (0.1)	N9 M1 P02.7	18 (0.8)	N9 M1 P02.7	1 (0.1)	N9 M1 P02.7	6 (0.3)
Obstetric haemorrhage	N9 M1 P02.1	10 (0.5)	N9 M1 P02.1	5 (0.2)	N9 M1 P02.1	1 (0.1)	N9 M1 P02.1	1 (0.1)	N9 M1 P02.1	0 (0.0)	N9 M1 P02.1	2 (0.1)
Hypertension	N9 M4 P00.0	50 (2.4)	N9 M4 P00.0	11 (0.5)	N9 M4 P00.0	2 (0.1)	N9 M4 P00.0	2 (0.1)	N9 M4 P00.0	0 (0.0)	N9 M4 P00.0	0 (0.0)
Anaesthetic complications	N9 M4 P04.0	1 (0.1)	N9 M4 P04.0	1 (0.1)	N9 M4 P04.0	0 (0.0)	N9 M4 P04.0	0 (0.0)	N9 M4 P04.0	0 (0.0)	N9 M4 P04.0	0 (0.0)

ICD-PM: International Classification of Diseases-perinatal mortality.

^a^ Since birth weights of > 1000 g at ≥ 28 weeks gestation were analysed, the only categories applicable for N9 were P07.1 (other low birth weight; 2051 deaths) and P07.3 (other preterm infants; 70 deaths); these groups were combined due to the small number in P07.3 to demonstrate how maternal codes can be applied for improved classification.

^b^ The Perinatal Problem Identification Program maternal condition category medical and surgical disorders includes cardiac disease (M4 P00.3), endocrine disease (M4 P00.9), gastrointestinal disease (M4 P00.9), central nervous system disease (M4 P00.9), respiratory disease (M4 P00.3), haematological disease (M4 P00.3), genitourinary disease (M4 P00.1), autoimmune disease (M4 P00.9), skeletal disease (M4 P00.9), psychiatric disease (M4 P00.9), neoplastic disease (M4 P00.9), or other medical and surgical disorders (M4 P00.9) using ICD-PM coding.

## Discussion

Here we show that ICD-PM coding improve consideration of maternal complication when classifying perinatal deaths. Previous research in South Africa reported that maternal complications were linked to around one half of all stillbirths and one quarter of early neonatal deaths.^13^ According to the South African classification system, 45.7% (8644/18 927) of stillbirths and 27.4% (2158/7883) of early neonatal deaths were classified as being linked to a maternal complication; this is equivalent to 40.3% (10 802/26 810) of all deaths. In contrast, our analysis of ICD-PM classifications identified a much higher proportion of maternal conditions for these outcomes. Maternal complications were associated with 59.0% (9208/15 619) of antepartum deaths, 89.0% of (3314/3725) intrapartum deaths and 79.6% (5945/7466) of neonatal deaths; this is equivalent to 68.9% (18 467/26 810) of all deaths.

We managed to classify all neonatal deaths with a primary cause of intrapartum asphyxia with an associated maternal condition using the ICD-PM codes, while the South African classification system only classified 17.4% (512/2942). Several subcategories such as labour-related intrapartum asphyxia, cord around the neck and others as outlined in Box 1 are classified according to the South African classification system as perinatal complications with a healthy mother. Using the ICD-PM system, however, these deaths can be correctly categorized as the result of a maternal condition. Antepartum haemorrhage, because of abruptio placentae or placenta praevia is considered a perinatal condition under the South African classification system, but classified as a maternal condition by the ICD-PM system.

We also show that ICD-PM coding improve consideration of timing of death. A recent systematic review found that 59% of globally reported stillbirths had no information regarding the timing of death,^15^ making the appropriate timing of interventions difficult to identify. Further, in some resource-poor settings the timing of a perinatal death may be the only piece of information captured. This information should therefore be a part of any classification system.^16^

The application of the ICD-PM coding system to our data revealed a significant burden of deaths occurring during the antepartum period. Further, more than a quarter of early neonatal deaths were due to low birth weight. This highlights the already established importance of investment in antenatal care to reduce perinatal mortality. The 2016 WHO antenatal care recommendations^17^ include an increased number of antenatal care contacts in the third trimester. In response to these recommendations and the increased number of third-trimester stillbirths observed when antenatal care visits had not been made during this period, the number of recommended antenatal care visits was changed in South Africa in April 2017.^18^

A commonly cited burden of perinatal mortality is prematurity and prematurity-related causes.^19^ However, simply identifying that prematurity is an important contributor to deaths gives no information regarding the optimal timing for interventions. From the ICD-PM classification, we see that 36.7% (1270; coded under A5, disorders related to fetal growth) of deaths due to prematurity (3426; the total of deaths classified as A5, I6 or N9) occurred during the antepartum period, and that 72.7% (923/1270) of these deaths were also related to a maternal complication. This information is invaluable to public health workers and policy-makers in targeting interventions; a heightened awareness of the causes of such deaths allows a focus on preterm-related issues, showing that both obstetric and neonatal interventions are required.

For implementing ICD-PM coding, systematic training of data administrators in the classification of deaths using ICD-PM will be required to ensure familiarity with the new system, as well as consistency across settings. Data administrators will also need to have access to clinicians to discuss cases that do not clearly fit a specific ICD-PM classification. In our experience, however, the ICD-PM system is both clinically relevant and easy to use; for example, the coder for this study does not have a clinical background. There was a high level of agreement between the coder and the verifying obstetrician, with differences encountered in only two cases: (i) premature rupture of membranes with chorioamnionitis (M1 P01.1 according to coder, M1 P02.7 according to obstetrician) and (ii) unexplained uterine death (A3 according to coder, A6 according to obstetrician). This demonstrates the feasibility in implementing the ICD-PM codes to existing data sets by administrators or allied health providers, in consultation with clinicians. Data administrators can be trained in the application of ICD-PM coding under the mentorship of clinicians, an advantage in low-resource settings.

We noted some specific issues with ICD-PM, including mutually exclusive categories, deaths which could be classified under two different ICD-PM codes, multiple contributing factors for cause of death, and causes of death not captured by the South African classification system but considered by ICD-PM codes (or vice versa). Examples of these issues and potential solutions are discussed in Table 4.

**Table 4:** Issues arising in implementing ICD-PM coding to South Africa’s Perinatal Problem Identification Program in the classification of perinatal deaths

Issue	Examples from implementation	Outcome implemented/potential solution
Mutually exclusive categories	A preterm birth where cause of death is premature rupture of membranes with chorioamnionitis could be classified as either M1 P02.7 fetus and newborn affected by chorioamnionitis, or M2 P01.1 fetus and newborn affected by preterm rupture of membranes	These deaths were classified under M1 P02.7 fetus and newborn affected by chorioamnionitis
Multiple contributing factors to cause of death	Abruptio placentae complicated by maternal hypertension can be classified as abruptio placentae or abruptio placentae with hypertension. ICD-PM can classify abruptio placentae as: fetal blood loss, fetus and newborn affected by other forms of placental separation and haemorrhage, abruptio placentae (A4 P50 M1 P02.1); or fetus or newborn affected by maternal hypertensive disorders (A4 P50 M4 P00.0). Here the coder must decide as to which is the most important maternal condition, that is, the abruptio placentae or hypertension	These deaths were coded as A4 P50 M1 P02.1 (abruptio placentae)
	Deaths due to antepartum haemorrhage where a maternal condition was also present can be classified with both; the defining cause of death (haemorrhage) is classified (ICD-PM) as a maternal condition rather than a fetal condition. The fetal condition is classified as P50 (fetal blood loss) and the maternal condition under abruptio placentae (M1 P02.1), placenta praevia (M1 P02.0) or twin-to-twin transfusion (M1 P02.3). Competing interests arise where there are multiple maternal conditions, such as sepsis, anaesthetic complications, hypertension, medical and surgical complications, or non-pregnancy-related infections in addition to abrupio placentae or placenta previa. The coder must decide whether to code under M4 or M1	Where no other maternal condition was present, antepartum haemorrhage was coded under M1. M4 was used for antepartum haemorrhage with another maternal condition also present
Two different ICD-PM codes for same cause of death	Unexplained intrauterine death could have been coded as either A3 or A6, which represent the same end cause of death (antenatal asphyxia)	These deaths were coded as A6, with no deaths being classified under A3
Conditions not captured in the Perinatal Problem Identification Program but included in ICD-PM	M3 P03.4 fetus and newborn affected by caesarean section delivery is not captured by the South African system: caesarean section delivery is not a classifiable cause of death, with some deaths captured under the maternal condition complications of anaesthesia or medical and surgical disorders	More detailed information for these categories would enhance the alignment of the existing data collection system to ICD-PM
	Birth trauma (I2) is not captured by the South African system: most deaths due to birth trauma are classified as traumatic assisted delivery or other cause of death not described in classification	More detailed information for these categories would enhance the alignment of the existing data collection system to ICD-PM
Perinatal Problem Identification Program maternal condition classifications too broad	For maternal conditions in the South African system, only lead categories can be linked to perinatal death (i.e. hypertension, obstetric haemorrhage, medical and surgical disorders); no specific details (e.g. proteinuric hypertension, eclampsia, chronic hypertension, etc.) can be linked	Improved linkage between perinatal cause of death and certain maternal conditions would allow more specific maternal ICD-PM codes to be applied
	For deaths related to other complications of labour and delivery (M3, other complications of labour and delivery), a large proportion of cases were classified as unspecified under the code P03.9 fetus and newborn affected by complication of labour and delivery, unspecified. In the South African system, these deaths were classified as labour-related intrapartum asphyxia with no further detail as to the exact labour-related maternal cause of these deaths	It may be possible to reduce the number of deaths falling under this unspecified category if South African mortality audits were able to capture more detailed information around maternal causes for complications of labour and delivery, such as those conditions falling under: M3 P03.1 fetus and newborn affected by other malpresentation, malposition, disproportion during labour and delivery; or P03.6 fetus and newborn affected by abnormal uterine contractions and conditions classifiable under O60–O75
High proportion of antepartum deaths classified as unspecified causes with no maternal complication (A6 M5)	Initially it appeared that ICD-PM coding was not sufficiently sensitive to identify the causes of these antepartum deaths accurately; however, these deaths were at the highest descriptive level in the South African system. No more information regarding the cause of death was available	These deaths were due to unexplained or unknown causes. There could be no improvement in the ICD-PM classification system that would reduce the number of deaths classified as A6 M5

ICD-PM: International Classification of Diseases-perinatal mortality.

As maternal and perinatal outcomes are closely related, both mother and infant benefit from intervention;^2^ this is particularly relevant in the management of hypertension and care during the intrapartum period.^3^^,^^20^^,^^21^ However, possible challenges exist with the application of the ICD-PM system to data sets which consider perinatal death and maternal condition separately, introducing issues in the integration of the two systems. The adaption of integrated perinatal and maternal data collection systems may be difficult in poorly resourced settings. For countries that do not have well established death classification systems, future developments could consider autopsy review categories aligned with ICD-PM codes for better consistency between death review and coding stages. For example, the South African classification system could be strengthened to align more closely to ICD-PM as described in Table 4.

In conclusion, by allowing for an increased recognition of the role of maternal condition and the timing of death in perinatal mortality, our conversion of an existing national perinatal mortality data set to ICD-PM codes enhanced our understanding of the data. This work is part of a larger work investigating perinatal deaths in South Africa and the required interventions.^18^^,^^22^ Our new classification of perinatal deaths could inform the allocation of resources and the timing of interventions. Adopting the ICD-PM coding system internationally would lead to a consistent global perinatal death classification system, which would create comparable data that could inform policy-makers globally.
